# Prevalence and risk factors of diabetes foot ulcers in Kano, northwestern Nigeria

**DOI:** 10.1186/s40842-023-00155-4

**Published:** 2023-11-14

**Authors:** Raliyatu Aliyu, Ibrahim D. Gezawa, Andrew E. Uloko, Mansur A. Ramalan

**Affiliations:** 1 Endocrinology, Diabetes, and Metabolism Unit, Department of Internal Medicine, Federal Teaching Hospital (FTH), Katsina, Nigeria; 2 Endocrinology, Diabetes, and Metabolism Unit, Department of Internal Medicine, Bayero University (BUK), Kano, Nigeria; 3 Endocrinology, Diabetes, and Metabolism Unit, Department of Internal Medicine, Aminu Kano Teaching Hospital (AKTH), Kano, Nigeria

**Keywords:** Diabetes foot ulcer, Diabetes, Prevalence, Risk factors, Nigeria

## Abstract

**Background:**

Foot complications account for more hospital admissions than any other diabetes mellitus (DM) complications with adverse outcomes being foot ulcers and amputation.

**Objective:**

To determine the prevalence and risk factors of diabetic foot ulcers in Kano, Northwestern Nigeria.

**Methods:**

A descriptive cross-sectional study was conducted in the diabetes outpatient clinics and medical and surgical wards of two hospitals in Kano, Nigeria. Data were collected on socio-demographic characteristics, type, and duration of DM. The study subjects were assessed for the presence of and risk factors for foot ulcers.

**Results:**

We recruited 394 patients with DM (163 males and 231 females) with a mean (SD) age and duration of DM of 50.8 ± 12.5 years and 7.72 ± 6.65 years respectively. Type 2 DM was present in 95% of the study subjects. Diabetic foot ulcer (DFU) was present in 57 (14.5%) of the patients. Risk factors associated with DFU assessed using univariate analysis were older age, longer duration of DM, presence of peripheral neuropathy (PN), peripheral arterial disease (PAD), diabetic retinopathy, nephropathy, foot deformities, previous DFU, and poor glycemic control. The independent determinants of DFU were previous DFU, foot deformities, retinopathy, PN, PAD, and poor glycemic control.

**Conclusion:**

DFU can be found in our setting and the predominant risk factors for DFU are common and remain unchanged in our environment. This study, therefore, buttresses the effect of early detection and treatment of DM in preventing the complications that arise from the disease.

## Introduction

The International Diabetes Federation (IDF) estimates that the number of people with diabetes mellitus (DM) will rise from 537 million in 2021 to 643 million by 2030 and to 783 million in 2045, with approximately 80% of this increase occurring in developing countries [1]. The age group most at risk is 40 – 59 years, which makes up the workforce group of any population. Over 19 million people have been estimated to suffer from DM in Africa, a figure that is expected to rise to approximately 28.6 million in 2035 and 47.1 million in 2045, with Nigeria having a large number of approximately 2.7 million people living with DM [1].

Diabetes foot ulcers may be defined as a group of disorders in which neuropathy, ischemia, and infection lead to tissue breakdown in the lower extremities of people with DM, resulting in morbidity and possibly amputation [2].

Foot ulcers are one of the most common complications of DM. It is estimated that 2.5% of persons with diabetes develop diabetic foot lesions each year, of which 14 – 24% will require amputation.3 Every 30 seconds, a lower limb is lost somewhere in the world due to DM, many of which are preventable, as 85% are preceded by foot ulcers [3].

Studies have reported that diabetic foot ulcers are one of the most common causes of morbidity and mortality in the hospital setting. Several studies have also reported that certain risk factors in patients with diabetes increase their tendencies to develop DFU [4–6].

We aimed in this study to determine if the burden of DFU in our setting is on the rise despite efforts to curtail the incidence of foot ulcers in Kano and Nigeria in general. Second, although there are known traditional risk factors for DFU, we also aimed to determine if these same risk factors increase the occurrence of foot ulceration in Kano, Nigeria, as documented in other parts of the world. This study will therefore contribute to the update on the burden of the disease and its associated risk factors as well as provide a basis for more awareness on the prevention of one of the most preventable complications of DM.

## Subjects, materials, and methods

### Study design

This is a multicentre cross-sectional study carried out in the diabetes outpatient clinics and wards of two major hospitals: Aminu Kano Teaching Hospital (AKTH) and Murtala Muhammad Specialist Hospital (MMSH) in Kano, Northwestern Nigeria.

The sample size was arrived at from previous studies on DFU. Four hundred patients with diabetes were recruited for the study. A systematic sampling method was used to select the study subjects. The study population was adults with type 1 or 2 DM who attended the Endocrine and Diabetes clinic, as well as those on admission in the medical and surgical wards of both hospitals (AKTH and MMSH) during the study period. The sampling frame was obtained from the average number of adult DM patients who attended each hospital monthly. This was 300 in AKTH and 900 in MMSH totaling 1200. Using proportionate allocation 30% of the patients, approximately 120, were selected from AKTH and 70% approximately 280, were selected from MMSH. The ratio of recruited subjects in AKTH and MMSH was approximately 1:2. The patient population in MMSH was larger because most patients prefer to seek medical care in this hospital because of the relatively low cost of medical care in MMSH compared to AKTH. The sampling fraction and interval were then calculated. The sampling fraction = calculated sample size/ sampling frame, which was equal to 400/1200 = 1/3. The sampling interval = reciprocal of sampling fraction = 3. During the selection of patients from each of the study sites, every eligible third patient who presented at the diabetes clinic and the inpatient wards was recruited after randomly selecting the first patient by balloting. The patients were then screened for the presence of DFU to determine its prevalence, and subsequently, those with DFU were compared with those without DFU to assess the risk factors for foot ulcers. All consenting adult patients with Type1 and 2DM who presented to the clinics, medical and surgical wards in both hospitals during the study period were included. We excluded those who declined consent, pregnant women, and those with other known causes of peripheral neuropathy (i.e. from drugs, myelopathies, and end-organ failure). Those who satisfied the inclusion criteria were recruited by the researcher until the required sample size was obtained. Four hundred subjects were recruited, but only 394 completed the study. Six of the study subjects breached the study protocol and were hence excluded from participating in the study. The study was carried out over 6 months.

**Fig. 1:**
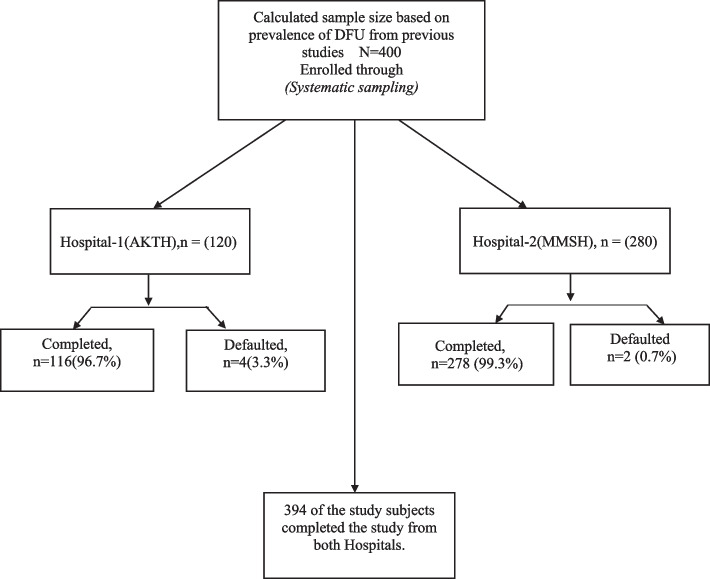
Flow chart showing the sampling technique used for subject’s recruitment. Abbreviations: AKTH = Aminu Kano Teaching Hospital; MMSH = Murtala Muhammad Specialist hospital

Figure 1 is a flow chart showing patients’ recruitment.

Approval for the study was obtained from the research and ethics committee of both Aminu Kano Teaching Hospital and Murtala Muhammad Specialist Hospital Kano.

### Study protocol/procedure

A structured questionnaire was used to obtain sociodemographic information, history of smoking, clinical variables including type of DM, duration of DM, history of complications of DM (hypertension, dyslipidemia, retinopathy, nephropathy, neuropathy, PVD), treatment modalities, history of foot ulcers and/ or amputations, duration of ulcer, glycemic control and presence of obesity. Anthropometric measurements (height and weight) were used to obtain body mass index (BMI). Also, the waist-hip ratio (WHR) was computed after conventional protocols for measuring the waist and hip circumferences were used.

Fundoscopic examination was conducted on the study subjects to determine the presence of diabetic retinopathy, which refers to the presence of preproliferative or proliferative retinopathy, in collaboration with an ophthalmologist.

We conducted foot examinations on each of the study subjects. The feet were inspected for skin changes, foot deformities (e.g., calluses, claw toes, hallux valgus, flat foot, and Charcot arthropathy), color changes, and the presence of ulcers. Wagner’s DFU classification system was used in grading foot ulcers. Evidence of previous foot surgery was also noted [7, 8]. The study subjects were told to bring their regular footwear during the prerecruitment process, and this footwear was then examined during the recruitment process to determine whether it was appropriate.

Neurological examinations were conducted on the study subjects as follows;

Pain Perception: This was assessed by the pinprick method; a new pin was used to prick each patient on the dorsum of the foot (on dermatomes L4, L5, and S1). The inability to feel the prick was considered positive for loss of pain perception [7].

Tactile Sensation: This was assessed twice with cotton wool applied on dermatomes L4, L5, and S1. Inability to feel it was considered positive for loss of touch sensation [7].

Temperature Sensation: This was improvised for and assessed with a cold tuning fork that was initially placed in a container with ice cubes and then applied to the dorsum of the foot. The inability to feel cold or no response was considered positive for loss of temperature sensation [7].

Joint position sense, protective sensation, and vibration perception were also examined using a standard protocol after explaining to the patient what to expect.

We conducted a vascular examination on the lower limbs of the study subjects, which included; pulsation of the dorsalis pedis and posterior tibialis artery identified by palpation. A hand-held Doppler probe together with a blood pressure cuff was used to determine the ankle-brachial pressure index (ABPI), which compares the systolic pressure in the upper and lower extremities. The ABPI was calculated as the systolic pressure on the leg divided by the systolic pressure in the arm. This was conducted using a standard protocol for measuring ABPI. For a foot with an ulcer, ischemic foot changes were noted, and ABPI was measured only in the leg without an ulcer. A normal ABPI is between 0.9 and 1.2. An index of less than 0.9 is abnormal and indicates the presence of obstruction, while 0.5 or less indicates a critical ischemic arterial disease. Values greater than 1.2 may indicate the presence of medial vessel calcification [7].

Definition of operational terms used in the study;

Hypertension was defined based on the JNC – 8 criteria as a positive history of hypertension, use of antihypertensive drugs, or blood pressure equal to or greater than 140/90 mmHg measured using a standard procedure [9].

Diabetic neuropathy was defined as the presence of symptoms. i.e. numbness, paraesthesia, and /or signs that include impaired VPT, touch, temperature, pain, and loss of joint position sense, and when 4 out of 10 sites at monofilament testing were not felt [7]. If one or more of the aforementioned was positive, it was deemed diagnostic [8].

Peripheral arterial disease (PAD) was said to be present if there was a history of symptoms and/or signs: intermittent claudication, rest pain, absence of 2 or more pedal pulses, and ABPI of < 0.9 or an ABPI of < 0.9alone [8].

Diabetic nephropathy was defined as the presence of Proteinuria/microalbuminuria taken on two separate urine samples on two separate occasions 3 months apart during the study period using Combi 10/Microalbustix [10].

Inappropriate footwear was defined as footwear that did not completely accommodate the feet, was highly heeled, or caused cramping of the toes [11].

Obesity was defined based on WHO guidelines as the body weight (kg) of an individual divided by the square of the height in meters, expressed in kg/m2. Subjects with BMI < 18.5 were classified as underweight, and those with a BMI of 18.5-24.9 were classified as having normal weight. Those with BMIs of 25.0-29.9 and ≥ 30.0 were classified as overweight and obese, respectively [12].

Dyslipidemia was defined using the adult treatment panel III (ATP III) guidelines when one or all of the following are found; 12 Total cholesterol > 200 mg/dl (5.2 mmol/l), LDL > 100 mg/dl (2.6 mmol/l), Triglycerides > 150 mg/dl (1.7 mmol/l) and HDL < 40 mg/dl (1.03 mmol/L) in men or < 50 mg/dl (1.30 mmol/L) in women.

Glycaemic control was defined based on the ADA [13] 2006 clinical recommendation for standards of medical care in diabetes. Good glycaemic control was defined as HbAlc < 7%, FPG = 4.4 – 7.2 mmol, and 2-hour post-prandial glucose ≤10 mmol/L.

### Statistical analysis

Data collected were analyzed using Statistical Package for Social Sciences (SPSS) version 21 (Chicago IL USA). Continuous variables were expressed as the mean (SD). Categorical data (variables) were expressed as proportions. Student’s t-test was used for the comparison of means, while the chi-square test (χ2) was used to compare proportions. Multivariate analysis, using logistic regression statistics, was used to determine the independent risk factors for DFU in the study population. In all cases, a *P* value < 0.05 was considered significant.

## Results

Of the 394 patients recruited for the study, 163 (41.4%) were males, while 231 (58.6%) were females. The mean ± (SD) age of the subjects was 50.8 ± 12.5 years. The mean ± (SD) duration of DM was 7.72 ± 6.25 years. More than half of the patients (303, 77%) were married, and 241 (61.2%) had formal education. Only 245 (62%) study subjects were gainfully employed. Most of the study subjects (373, 94.7%) had type 2 DM. Table 1 below shows the sociodemographic characteristics of the study subjects.

**Table 1:** Sociodemographic characteristics of study subjects

Age Group (yrs)	Frequency n (%)
15 – 24	13 (3.3)
25 - 34	23 (5.9)
35 - 44	76 (19.3)
45 - 54	125 (31.7)
55 - 64	97 (24.6)
≥65	60 (15.2)
Mean(SD) Age	50.8 ± 12.5 yrs
Sex	
Male	163 (41.4)
Female	231 (58.6)
Marital Status	
Single	20 (5.0)
Married	303 (77.0)
Divorced	6 (2.0)
Widowed	65 (16.0)
Educational Status	
Quranic	153 (38.8)
Primary	66 (16.8)
Secondary	67 (17.0)
Tertiary	108 (27.4)
Occupational status	
Unemployed	151 (38.3)
Farmers	6 (1.5)
Traders	113 (28.7)
Artisan	37 (9.4)
Civil servants	50 (12.7)
Professionals	37 (9.4)
Type of DM	
Type 1	21 (5.3)
Type 2	373 (94.7)
Others	0 (0.0)
Duration of DM	
Mean (SD)	7.72 ± 6.25 (100.0)

**Fig. 2:**
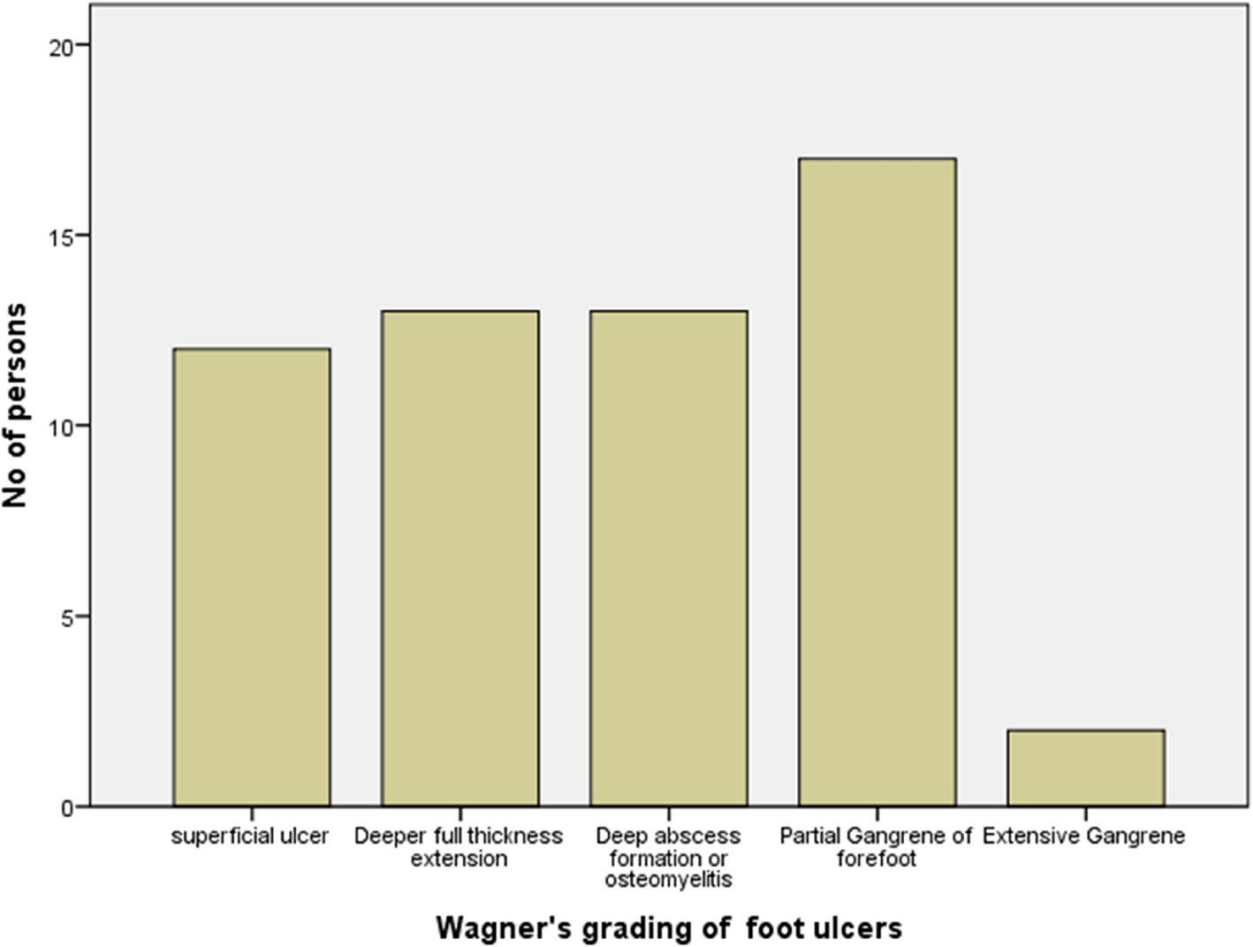
Wagner’s classification of foot ulcers of study subjects

The prevalence of DFU among the study subjects was 57 (14.5%). There were more females with foot ulcers (*n* = 33, 57%). The Wagner grading system of DFU is shown in Fig. 2. Twenty-three (46%) of the subjects presented with Wagner grades 2 and 3 while 17 (30%) had grade 4 DFU. Forty-five (79%) of the foot ulcers were purely neuropathic, 10 (17.5%) had neuro ischemic ulcers while 2(3.5%) were purely ischemic.

The independent determinants of foot ulcers were a previous history of foot ulceration, the presence of foot deformities, diabetic retinopathy, peripheral neuropathy, peripheral vascular disease, and poor glycemic control, as shown in Table 2.

**Table 2:** Independent Risk Factors for Diabetic Foot Ulcers (DFU)

Variable	Odds Ratio	95% CI	*P*value
Older Age	2.056	0.966 – 2.404	0.06
Long duration of DM	0.914	0.442 – 1.889	0.81
Past history of DFU	2.205	1.112 – 4.370	0.02*
Presence of foot deformities	3.369	1.678 – 6.762	0.001*
PN	6.245	2.240 – 17.41	0.001*
PVD	2.753	1.295 – 5.855	0.001*
Retinopathy	1.632	1.109 – 2.404	0.01*
Proteinuria	0.843	0.408 – 1.742	0.64
Poor glycemic control	0.362	0.165 – 0.796	0.01*

*DFU* Diabetes Foot Ulcer, *PN* Peripheral Neuropathy, *PVD* Peripheral Vascular Disease, glycemic control is good when HbA1c < 7% and poor if HbA1c > 7%, *CI* Confidence Interval, * significantly *p*-value.

Other risk factors for DFU were assessed as shown in Table 3. The potential risk factors associated with DFU from the study were older age (*p* = 0.030), longer duration of DM (*p* = < 0.001), presence of foot deformities (*p* = < 0.001), previous history of foot ulcerations (*p* = < 0.001) and poor glycemic control (*p* = 0.001). Microvascular complications of DM, such as PN (*p* = < 0.001), PVD (*p* = < 0.001), retinopathy (*p* = < 0.001), and proteinuria (*p* = 0.026), were also found to be associated with foot ulcers. However, sex, marital status, social class, smoking, BMI, WC, inappropriate footwear, and dyslipidemia were not significantly associated with DFU (*p* ≥ 0.05).

**Table 3:** Risk Factors for diabetic foot ulcers (DFUs) among the study subjects

Variables	DFU *n* = 57	WITHOUT DFU *n* = 337	Test statistics *X*^*2*^	*P* VALUE
Socio – demographic				
Gender				
Male	24 (14.6)	140 (85.4)	0.006	0.936
Female	33 (14.3)	197 (85.7)		
Age				
Young	9 (8.3)	100 (91.7)		
Middle age	40 (16.4)	204 (83.6)	4.696	0.030*
Elderly	8 (19.5)	33 (80.5)		
Marital status				
Single	2 (9.5)	19 (91.5)		
Married	37 (12.2)	267 (87.8)	0.438	0.508
Divorced	4 (57.1)	3 (42.9)		
Widowed	14 (22.6)	48 (77.4)		
Educational status				
Informal	25 (16.4)	127 (83.6)	0.784	0.376
Formal	32 (13.2)	210 (86.8)		
Socio – Class				
1	12 (15.0)	68 (85.0)		
2	3 (27.2)	8 (72.7)	0.389	0.532
3	11 (11.6)	84 (88.4)		
4	5 (10.4)	43 (89.6)		
5	26 (16.3)	134 (83.7)		
Type of DM				
Type 1	3 (12.5)	18 (87.5)	0.000	0.989
Type 2	54 (14.5)	319 (85.5)		
Others	0 (0.0)	0 (0.0)		
Duration of DM (yrs)				
Short term	15 (9.7)	139 (90.3)	3.853	< 0.001*
Medium term	17 (12.3)	121 (87.7)		
Long term	25 (24.5)	77 (75.5)		
Cigarette Smoking				
Yes	3 (25.0)	9 (75.0)	1.111	0.292
Ex – smoker	6 (31.6)	13 (68.4)		
No	48 (13.2)	315 (86.8)		
CLINICAL VARIABLES				
Hypertension				
Present	29 (14.0)	177 (86.0)	0.053	0.818
Absent	28 (14.9)	160 (85.1)		
Peripheral Neuropathy				
Present	49 (24.6)	150 (75.4)	33.51	< 0.001*
Absent	8 (4.1)	187 (95.9)		
Peripheral vascular disease				
Present	19 (39.6)	29 (60.4)	27.79	< 0.001*
Absent	38 (11.0)	308 (89.0)		
Diabetic Retinopathy (*n* = 258)				
Present	18 (24.0)	57 (76.0)	14.31	< 0.001*
Absent	13((7.1)	170 (92.9)		
Proteinuria				
Present	38 (18.2)	171 (81.8)	4.964	0.026*
Absent	19 (10.3)	166 (89.7)		
Foot Deformities				
Present	36 (24.2)	113 (75.8)	18.19	< 0.001*
Absent	21 (8.6)	224 (91.4)		
Previous foot ulcer				
Yes	24 (29.3)	58 (70.7)	18.33	< 0.001*
No	33 (10.6)	279 (89.4)		
Previous Amputation				
Yes	3 (37.5)	5 (62.5)	3.501	0.06
No	54 (14.0)	332 (86.0)		
Inappropriate footwear				
Yes	51 (15.7)	274 (84.3)	2.252	0.133
No	6 (8.7)	63 (91.3)		
LABORATORY VARIABLES				
Poor Glycemic control				
HbA1c (%)				
Yes	49 (18.4)	218 (81.6)	10.10	0.001*
No	8 (6.3)	119 (93.7)		
Dyslipidemia				
Yes	25 (16.7)	125 (83.3)	0.945	0.331
No	32 (13.1)	212 (86.9)		

Young ≤45 years, Middle-age > 45 < 64 years, Elderly≥65 years**,** Short-term< 5 yrs., Medium term = 5-10 yr Long-term> 10 yrs., Good glycemic control HbA1c < 7.0%, Poor glycemic control HbA1c ≥7.0%, Proteinuria present > 30 mg/l, Proteinuria absent < 30 mg/l, Retinopathy present = pre proliferative/ proliferative retinopathy, absent = no pre proliferative /proliferative retinopathy *p-value statistically significant.

The mean clinical and laboratory values of subjects with and without DFU are shown in Table 4 below. Subjects with DFU were older (*p =* 0.033), had a longer duration of DM (*p* <  0.001), had evidence of PAD and PN (*p <* 0.001), and had poor short and long-term glycemic control (*p <* 0.001) compared with those without DFU. Anthropometric measurements (BMI, WC, and WHR), type of DM, and dyslipidemia were not significantly different between subjects with and without DFU (*p* ≥ 0.05).

**Table 4:** Clinical and biochemical characteristics of subjects with and without DFU

	DFU		*p* value
	Present	Absent	
Clinical Characteristics			
Mean Age (years)			
Males	53.56 ± 9.56	51.43 ± 13.76	0.462
Females	55.23 ± 13.34	49.53 ± 12.13	0.012*
All	54.49 ± 11.18	50.32 ± 12.51	0.033*
Mean DM Duration (years)	10.62 ± 8.02	7.23 ± 5.78	< 0.001*
Type of DM			
Type 1	3 (5.3)	18 (5.3)	
Type 2	54 (94.7)	319 (94.7)	0.945
Others	0 (0.0)	0 (0.0)	
Mean BMI (kg/m^2^)			
Males	23.59 ± 11.27	26.24 ± 5.11	0.069
Females	25.20 ± 8.77	28.23 ± 6.03	0.013*
All	24.50 ± 9.88	27.56 ± 5.79	0.497
Mean ± SD WC (cm)			
Males	95.72 ± 18.04	97.81 ± 13.66	0.050
Females	100.75 ± 13.05	98.77 ± 12.74	0.894
All	98.54 ± 15.50	98.43 ± 13.06	0.951
Mean ± SD WHR			
Males	0.92 ± 0.08	0.95 ± 0.08	0.078
Females	0.95 ± 0.06	0.93 ± 0.07	0.099
All	0.94 ± 0.07	0.94 ± 0.08	0.916
Mean ± SD BP (mmHg Systolic	132.63 ± 22.24	132.36 ± 19.40	0.605
Diastolic	82.46 ± 11.99	83.64 ± 11.77	0.943
ABPI n (%)			
< 0.9	12 (21.1)	17 (5.0)	< 0.001*
0.9 – 1.3	27 (47.4)	242 (71.8)	
>1.3	18 (31.6)	72 (21.4)	
VPT n (%)			
<25	10 (17.5)	235 (69.7)	< 0.001*
>25	47 (82.5)	102 (30.3)	
Biochemical characteristics			
Mean ± SD FPG			
Males	11.03 ± 3.49	8.82 ± 3.56	0.001*
Females	9.94 ± 3.85	8.90 ± 3.72	0.143
All	10.42 ± 3.70	8.67 ± 3.68	0.001*
Mean ± SD 2HPP			
Males	13.03 ± 3.50	12.06 ± 3.97	0.260
Females	12.99 ± 3.54	11.43 ± 3.92	0.035*
All	13.01 ± 3.54	11.65 ± 3.94	0.014*
Mean ± SD HbA1c (%)			
Males	10.81 ± 3.24	8.94 ± 3.51	0.015*
Females	10.94 ± 3.51	8.65 ± 3.01	< 0.001*
All	10.88 ± 3.10	8.75 ± 3.12	< 0.001*
Mean Plasma Lipids (mg/dl)			
T Cholesterol	200.4 ± 81.05	216.18 ± 82.79	0.165
HDL	51.78 ± 22.78	51.45 ± 22.78	0.928
TG	138.17 ± 64.75	153.23 ± 74.75	0.150
LDL	121.42 ± 61.91	134.23 ± 60.94	0.144
TC/HDL	4.67 ± 4.45	4.53 ± 1.52	0.675

Data are expressed in Mean ± SD, *BMI* Body mass index, *WC* Waist circumference, *WHR* Waist hip ratio, *BP* Blood pressure, *FPG* Fasting plasma glucose, *2HPP* 2 hrs post prandial, * statistically significant.

## Discussion

Diabetic foot ulcers constitute one of the most devastating consequences of DM with an increase in morbidity and mortality. Regular screening of the feet, early detection of at-risk feet, and appropriate treatment of foot ulcers could prevent 85% of amputations. This study will therefore contribute to the growing knowledge of the prevalence and risk factors of DFU to improve the care of DFU in Nigeria.

The prevalence of diabetic foot ulcers in this study was 14.5%. This prevalence compares well with the 12.5% prevalence reported by Uloko et al [14] in an earlier study. When compared to figures observed in different hospital-based studies in Nigeria (0.9 – 32.2%), 5 [14–16], the prevalence in the index study is also high. Possible explanations for the varying prevalence in these Nigerian studies may include differences in the study methods deployed, the study period, and the sociodemographic characteristics of the various populations, among other reasons. Studies from Cameroun [17] and Tanzania [18] also reported similar prevalence rates of 13 and 15%, respectively. A higher prevalence of 19% was, however, reported from Burkina faso18. A larger sample size was used in our study in contrast to the latter study, which could explain the difference.

Studies from the UK and the Middle East have found lower prevalence rates of DFU among their subjects [19–21]. Higher socioeconomic status, quality of care, health insurance coverage, and differences in health policies, may account for the lower prevalence of DFU in those populations.

The risk factors for DFU identified using univariate analysis in this study include older age, longer duration of DM, presence of PN, PVD, diabetic retinopathy and Proteinuria, presence of foot deformities, previous history of foot ulceration, and poor glycemic control.

The finding of older age as a risk factor for DFU in this study concurs with reports from studies in Tanzania [22] and Bahrain [19]. Type 2 DM is more common among the elderly, in whom both microvascular and macrovascular complications of the disease are more likely, thereby predisposing them to the development of foot ulceration. The prevalence of DFU in this study increased steadily with the increasing duration of diabetes. Other studies have reported similar findings [19, 23–25]. However, Bokyo et al [26] observed that a longer duration of DM did not increase susceptibility to foot ulceration among their patients.

Peripheral neuropathy, PVD, and previous foot ulcers were also found to be significantly associated with the occurrence of DFU in our study. This is consistent with reports from other studies [5, 26–28]. Both peripheral neuropathy and PVD are cardinal events in the pathway to foot disorders. The role of PN in the increased occurrence of DFU results from motor, sensory, and autonomic neuropathy, which leads to drying of the skin and its subsequent breakdown, increased pressure, and foot deformities culminating in ulcer formation. Peripheral arterial disease, on the other hand, causes decreased luminal blood flow, thereby preventing wound healing and leading to gangrene and amputation.

Proteinuria and retinopathy were also observed to be associated with foot complications in this study, which is similar to findings in China [27] and the USA26 but in contrast with reports by Mostapha et al [23] who observed that retinopathy was not associated with foot complications.

Studies conducted by Ogbera et al. [5], Bokyo et al. [26] and Ahmad et al [28] showed that the presence of foot deformities and poor glycemic control increased the tendency to develop DFU.

Cigarette smoking, whether current or past, was not significantly associated with DFU in this study, which was also documented in previous studies by Hellar et al [22] and Bokyo et al [26]. However, in contrast with this study, Yekta et al [29] and Al – Mahroos et al [19] reported that cigarette smoking was significantly associated with DFU. The lack of association between cigarette smoking and DFU in this study may be due to the low rate of cigarette smoking among our subjects.

Contrary to our findings, Mostapha et al [24] and Yekta et al [29] observed that low educational status and hypertension were associated with foot ulcers. The low rate of hypertension among our subjects with DFU may explain the lack of association observed.

Although the majority of our subjects wore inappropriate footwear, a trivial cause of foot disorders in the presence of PN, it was not associated with DFU in the index study.

On multivariate logistic regression analysis, we found PN, PVD, diabetic retinopathy, and the presence of foot deformities to be independent risk factors for DFU in this study. Other risk factors included previous foot ulcers and poor glycemic control. Similar findings have been reported previously [19, 27, 29]. Neither retinopathy nor proteinuria was found to be associated with DFU after subjecting both variables to logistic regression in the index study. The relatively small sample size employed in our study may explain these findings.

The clinical characteristics that were significantly associated with DFU in our study were older age, longer duration of DM, PVD, PN, and poor glycemic control. Subjects with DFU were significantly older than those without DFU in this study. This compares well with studies from Lagos [5], Iran [29], Saudi Arabia [21], and the Netherlands [25] but in contrast to what was observed in Ile – Ife, Southwestern Nigeria [30], where subjects with and without foot ulcers were comparable in terms of their age, possibly because of the small sample size used in that study.

A longer duration of DM was also a significant clinical variable associated with DFU in the index study. This was also found in previous studies, which showed that subjects with DFU had a longer duration of DM compared to those without [19, 23, 24, 31]. It is well known that the longer the duration of DM, the more the tendencies to develop micro- and macrovascular complications of DM and the higher the risk of developing foot complications.

Generally, subjects with DFU in our study had significant PVD/PN compared with those without DFU as observed in previous studies [5, 26–28].

The glycemic control of the study subjects in our study was found to be poorer in those with DFU. This compares well with observations from Lagos, Kenya, Bahrain, Poland, and Europe [5, 22, 32–34] but differs from reports from Ile Ife Southwestern Nigeria30 and Saudi Arabia [23], which showed that glycemic control was comparable between the two groups. Poor glycemic control predisposes patients to DFU and prevents early wound healing.

To the best of our knowledge, this is the largest study on the prevalence and risk factors for DFU in Kano, northwestern Nigeria. We hope that this study will provide additional information on the subject, to improve the care of DFUs in our setting and the country at large.

We had several limitations. The study was a cross–sectional design involving only patients who presented to the hospitals, thereby underestimating the number of patients with DFU. In addition, we were unable to undertake electrophysiological nerve conduction and angiographic studies (due to cost considerations), which would have further confirmed the presence of PN and PVD, respectively.

Our study shows that known risk factors for DFUs are common amongst our patients so presently a diabetic foot working group and diabetes foot initiative are now on board with intent to enhance the quality of care for persons with diabetes mellitus so as to minimize adverse complications that arise from the disease. Collaboration is also needed in smaller communities where access to specialized healthcare services is limited to identify, prevent and provide care for individuals at risk of foot ulcers.

Though only a few of our patients with DFU had grade 5 Wagner’s classification of foot ulcers, a majority had grades 2,3 and 4 which if not properly managed can easily progress with disastrous consequences. To avoid such undesirable results and other complications of DM, advocacy groups should actively work with legislators and healthcare professionals to develop national guidelines and training programs on how to diagnose and treat diabetes mellitus. It is also necessary to actively treat modifiable risk factors like dyslipidemia, hypertension, and obesity to minimize the risk factors for DFUs. Also encouraged is a comprehensive foot check at least once a year. Every visit should include a foot inspection for patients with sensory loss or previous ulceration as well as an ABPI examination, especially for those who are at risk of developing PAD.

## Conclusion

Diabetic foot ulcers can be found in Kano, as in other parts of the country. The predominant risk factors associated with DFU in this study were older age, longer duration of DM, PN, PAD, diabetic retinopathy, proteinuria, previous history of DFU, presence of foot deformities, and poor glycemic control. The independent determinants of foot disease were previous history of DFU, presence of foot deformities, PN, PAD, diabetes retinopathy, and poor glycemic control. We recommend larger community-based studies to determine the prevalence of DFU, its risk factors, and possible treatment outcomes of foot ulcers in persons with DM.
